# Reduced postoperative inflammation and blood loss after robotic arm‐assisted unicompartmental compared with total knee arthroplasty: A propensity score‐matched analysis

**DOI:** 10.1002/ksa.70212

**Published:** 2025-12-07

**Authors:** Dirk Müller, Florian Pohlig, Benjamin Schloßmacher, Vincent Lallinger, Michael T. Hirschmann, Rüdiger von Eisenhart‐Rothe, Igor Lazic

**Affiliations:** ^1^ Department of Orthopaedic Surgery, School of Medicine, Klinikum Rechts der Isar Technical University of Munich Munich Germany; ^2^ Schoen Clinic Munich Munich Germany; ^3^ Department of Orthopaedic Surgery and Traumatology Kantonsspital Baselland (Bruderholz, Liestal, Laufen) Bruderholz Switzerland

**Keywords:** blood loss, C‐reactive protein (CRP), matched‐pairs analysis, robotic arm‐assisted total knee arthroplasty (raTKA), robotic arm‐assisted unicompartmental knee arthroplasty (raUKA), systemic inflammatory response

## Abstract

**Purpose:**

Robotic arm‐assisted unicompartmental knee arthroplasty (raUKA) offers potential advantages over robotic arm‐assisted total knee arthroplasty (raTKA), but its biological benefits remain unclear. Superior early recovery after raUKA may be related to reduced systemic inflammation and blood loss. It was hypothesised that raUKA would elicit a lower systemic inflammatory response and result in reduced perioperative blood loss compared with raTKA.

**Methods:**

Patients undergoing raUKA with the Mako® system were 1:1 propensity score‐matched to raTKA patients based on age, gender, body mass index, and ASA score, yielding two balanced cohorts (*n* = 162 each). Blood samples were collected preoperatively, 6 h postoperatively, and on postoperative Days 3 and 5. Primary outcomes were C‐reactive protein (CRP) and white blood cell (WBC) levels; secondary outcomes included operative time and calculated blood loss.

**Results:**

CRP increases were significantly lower after raUKA on Day 3 (1.6 vs. 4.6 mg/dL, *p* < 0.001) and Day 5 (0.8 vs. 3.7 mg/dL, *p* = 0.031). WBC counts were lower at 6 h in raUKA (3.8 vs. 4.5 G/L, *p* = 0.041) but not significantly different on Days 3 or 5. Median operative time was shorter with raUKA (81 vs. 95 minutes, *p* < 0.001). Haemoglobin decrease and estimated blood loss were reduced in raUKA compared with raTKA (532 vs. 669 mL, *p* < 0.001).

**Conclusion:**

This is the first study to directly compare inflammatory response and blood loss between raUKA and raTKA. raUKA was associated with lower CRP and early WBC elevations, reduced blood loss, and shorter operative times. These results provide clinically relevant biomarker reference values and highlight perioperative advantages of raUKA. Collectively, they support raUKA as a less invasive alternative to raTKA in appropriately selected patients.

**Level of Evidence:**

Level III, retrospective comparative study.

**Abbreviations**
ASA: American Society of Anesthesiologists
BMI: body mass index
CRP: C‐reactive protein
Hb: haemoglobin
raTKA: robotic arm‐assisted total knee arthroplasty
raUKA: robotic arm‐assisted unicompartmental knee arthroplasty
TKA: total knee arthroplasty
UKA: unicompartmental knee arthroplasty
WBC: white blood cell count

## INTRODUCTION

Robotic arm‐assisted unicompartmental knee arthroplasty (raUKA) has been associated with several advantages over conventional manual techniques, including improved implant positioning and limb alignment [2, 3, 5, 9, 14, 21, 24, 45], reduced postoperative pain and hospital stay [6, 16, 21], superior early functional outcomes [4, 5, 12, 16, 21], more physiological gait patterns [29] and lower revision rates [2, 6, 27].

Compared with robotic arm‐assisted total knee arthroplasty (raTKA), raUKA may represent a less invasive option. It has been linked to faster recovery, particularly during the first postoperative year [26]. However, the biological response to raUKA, and in particular its postoperative inflammatory profile, remains largely unexplored.

Inflammatory biomarkers such as C‐reactive protein (CRP) and white blood cell (WBC) count are widely used to monitor recovery and identify complications after orthopaedic surgery [39]. After total knee arthroplasty (TKA), CRP typically peaks on postoperative Days 2–3 [15, 33, 34, 38] and returns to baseline within 2–4 weeks [15, 34]. Similarly, a transient leukocytosis is frequently observed in the early postoperative period after TKA [8]. Prior studies have shown that conventional unicompartmental knee arthroplasty (UKA) elicits a lower inflammatory response than TKA [1, 19, 37, 42]. Yet reported postoperative CRP values vary considerably, ranging from 1.3 to 46.6 mg/dL for UKA and 3.9 to 66.4 mg/dL for TKA, highlighting the need for more precise characterisation.

To date, no study has specifically evaluated the inflammatory response following raUKA. Similarly, blood loss has been reported to be lower after conventional UKA compared with TKA [28, 36], but evidence regarding blood loss after raUKA is limited [17, 23].

It was therefore hypothesised that raUKA would be associated with a lower postoperative inflammatory response and reduced blood loss compared with raTKA. The aim of this study was (1) to characterise inflammatory markers and blood loss following raUKA, and (2) to compare these findings with those observed after raTKA.

## MATERIALS AND METHODS

### Patient selection and matching procedure

Between October 2019 and June 2024, a total of 780 robotic arm‐assisted knee arthroplasties were performed at our university hospital using the Stryker Mako® robotic system (Stryker, Kalamazoo, USA). Exclusion criteria were bilateral knee arthroplasty, isolated patellofemoral joint arthroplasty, postoperative surgical site infection, infection of other organs, and haematologic disorders. After applying these criteria, 576 patients who underwent raTKA and 162 patients who underwent raUKA were eligible for inclusion in this retrospective study.

To reduce confounding, 1:1 propensity score matching was performed without replacement. Matching was based on gender, age, body mass index (BMI), and ASA score. A caliper width of 0.2 standard deviations of the logit of the propensity score was applied, and optimal matching was achieved using the Hungarian algorithm. This resulted in two balanced cohorts of 162 patients each.

**Figure 1:**
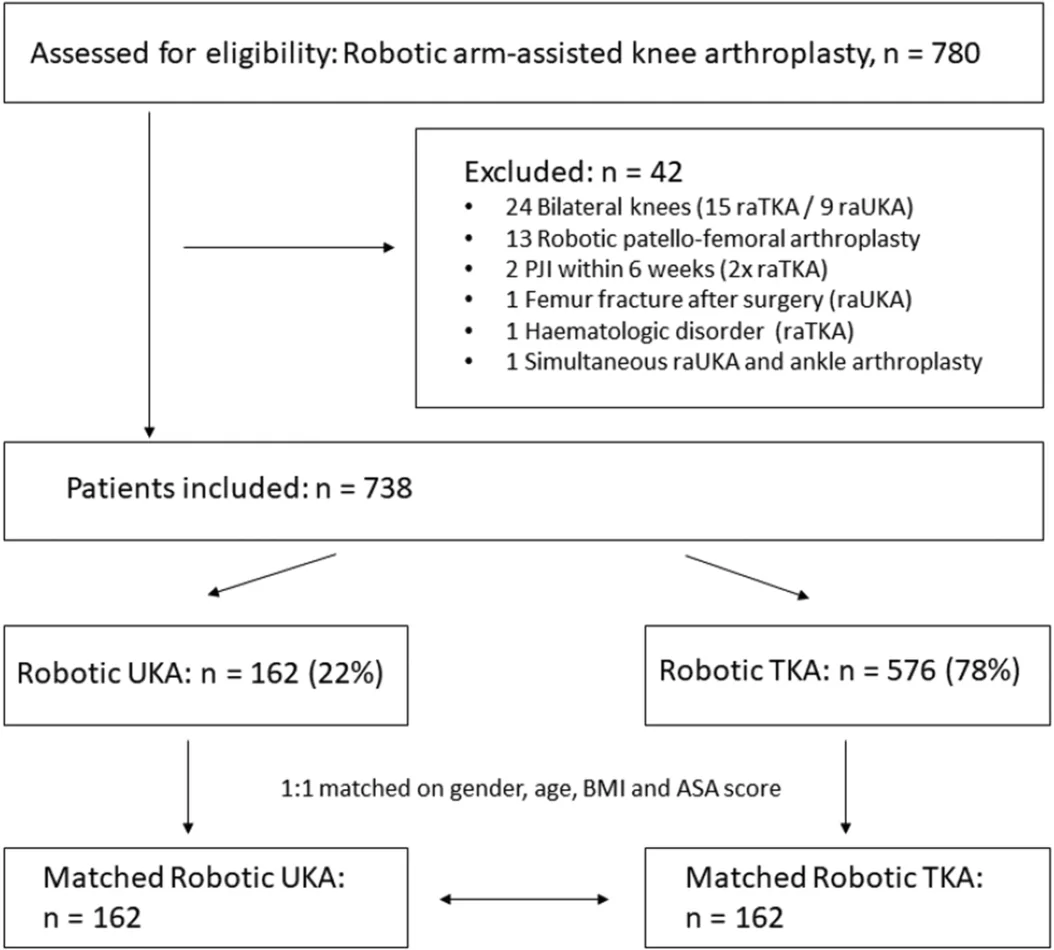
Flowchart of patient selection and matching procedure. Abbreviations: raTKA, robotic arm‐assisted total knee arthroplasty; raUKA, robotic arm‐assisted unicompartmental knee arthroplasty.

The adequacy of matching was evaluated using standardised mean differences (SMDs) and appropriate paired statistical tests (Wilcoxon signed‐rank or McNemar's exact test). All matched variables showed negligible imbalance (SMD < 0.1, *p* > 0.05), confirming the effectiveness of the matching procedure. The patient selection and matching flowchart is presented in Figure 1, and baseline characteristics are summarised in Table 1.

**Table 1:** Patient characteristics.

	Robotic UKA (*n* = 162)	Robotic TKA (*n* = 162)	*p* value	SMD
Gender (female/male, %)	44/56	49/51	0.382	0.099
Age (years at surgery, median, Q1–Q3)	65 (59–72)	65 (58–72)	0.522	−0.007
BMI (kg/m², median, Q1–Q3)	28.4 (25.9–31.2)	28.3 (25.0–31.2)	0.798	0.060
ASA (I–II/III–IV,%)	85/15	87/13	0.711	0.054
Kellgren and Lawrence Grade (III/IV,%)	77.2/22.8	76.5/23.5	0.900	–
Laterality (left/right, %)	56/44	49/51	–	–
Crutiate retaining/posterior stabilised (%)	–	2/98	–	–
Patella resurfacing (%)	–	78	–	–
Medial/lateral UKA (%)	99/1	–	–	–

*Note*: All matched variables demonstrated negligible imbalance (SMD < 0.1, *p* > 0.05).

Abbreviations: ASA, American Society of Anesthesiologists; BMI, body mass index; SMD, standardised mean differences; TKA, total knee arthroplasty; UKA, unicompartmental knee arthroplasty.

### Surgical technique

For raUKA, the Mako® robotic system was used in combination with the Restoris® MCK partial knee implant (Stryker, Kalamazoo, USA). raTKA was performed using the Triathlon® total knee system (Stryker, Kalamazoo, USA). Preoperatively, all patients received 1 g tranexamic acid and 1.5 g cefuroxime intravenously. No tourniquet was used. All procedures were performed by four senior arthroplasty surgeons, each certified by the German EndoCert certification system and trained and certified by Stryker for the Mako® robotic system.

Indications for raUKA included Kellgren–Lawrence grade ≤ 2 in the non‐operated compartments, intact anterior cruciate ligament, and medial and lateral ligamentous stability. UKAs were implanted according to a kinematic alignment principle [35], with preoperative planning for optimal positioning using the Mako® software to restore the native joint line while accounting for cartilage wear. Intraoperatively, implant positions were fine‐tuned to achieve a balanced 1–2 mm gap throughout the range of motion. In the raTKA group, a functional alignment strategy was applied as described by Lustig et al. [25].

All implants were fixed using vacuum‐mixed bone cement containing gentamicin (Palacos R + G, Heraeus, Hanau, Germany). No surgical drains were used. After capsule closure, 1.5 g tranexamic acid was injected intra‐articularly. Thromboprophylaxis consisted of apixaban 2.5 mg twice daily for 2 weeks.

### Blood samples

Blood samples were collected preoperatively, 6 h postoperatively, and on postoperative Days 3 and 5. White blood cell count (WBC, ×10⁹/L) and haemoglobin concentration (Hb, g/dL) were analysed using the Sysmex XE‐5000 system (Sysmex, Germany). C‐reactive protein (CRP, mg/dL) was measured using the Cobas 8000 modular analyser (Roche, Germany). All parameters (CRP, WBC, Hb) were recorded to one decimal place.

Statistical significance of postoperative changes in CRP and WBC was assessed relative to baseline by calculating the difference between preoperative and corresponding postoperative values. Haemoglobin loss was calculated as the difference between preoperative (Hb_pre_) and postoperative (Hb_post_) concentrations. Intraoperative blood volume (BV) was estimated using the Nadler formula [31]:

- For males: BV (mL) = 1000 × (0.3669 × height (m)^3^ + 0.03219 × weight (kg) + 0.6041)
- For females: BV (mL) = 1000 × (0.3561 × height (m)^3^ + 0.03308 × weight (kg) + 0.1833)

The estimated blood loss (EBL) was calculated based on the haemoglobin balance method [13]:

$$\text{EBL}\left(\text{mL}\right)=\text{BV}\times \left({\text{Hb}}_{\text{pre}}-{\text{Hb}}_{\text{post}}\right)/{\text{Hb}}_{\text{pre}}\text{.}$$

### Ethical approval

This study was approved by the institutional review board of the local Ethics Committee of Technical University of Munich (IRB approval number 714/20 S). Written informed consent was obtained from all participants prior to inclusion.

### Statistical analysis

Statistical analyses were performed using SPSS version 29 (IBM Corp., Armonk, NY, USA). Normality of continuous variables was assessed with the Shapiro–Wilk test. Normally distributed variables are reported as mean ± standard deviation (SD), and non‐normally distributed variables as median (Q1–Q3). Between‐group comparisons of continuous variables were performed with the paired Student's *t*‐test or the Wilcoxon signed‐rank test, as appropriate. Categorical variables are presented as frequencies and percentages and compared using McNemar's exact test. A *p*‐value ≤ 0.05 was considered statistically. The effect size (*r*) was calculated as *r* = *Z*/√*N*, where *Z* is the standardised Wilcoxon statistic and *N* is the number of paired observations.

## RESULTS

**Figure 2:**
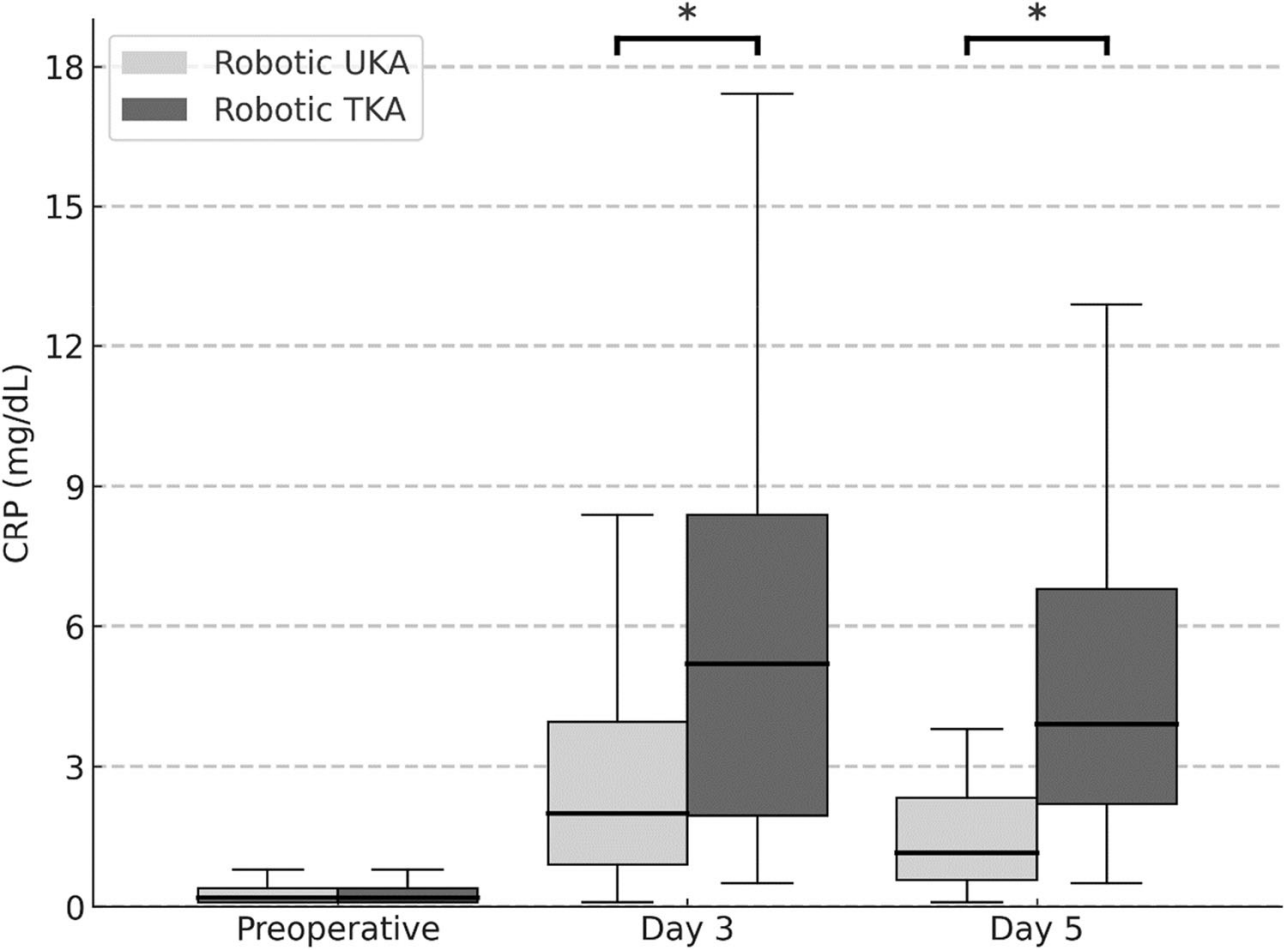
C‐reactive protein (CRP) levels (median, mg/dL) over time in robotic unicompartmental knee arthroplasty (raUKA) and total knee arthroplasty (raTKA). *p < 0.05. For the statistical analysis, postoperative CRP values were adjusted by subtracting the preoperative baseline value.

CRP levels peaked on postoperative Day 3 in both groups. On postoperative Days 3 and 5, CRP levels were significantly lower in the raUKA group compared with the raTKA group (Figure 2, Table 2).

**Table 2:** Summary of operative time, ∆ CRP and ∆ WBC, as well as haemoglobin decrease and calculated blood loss for robotic unicompartmental and total knee arthroplasty.

	Robotic UKA	Robotic TKA	*p* value	Effect size
Operative time (minutes), median (Q1–Q3)	81 (73–90), *n* = 162	95 (84–104), *n* = 162	***p*** < **0.001**	*r* = −0.50
∆ CRP Day 3 (mg/dL), median (Q1–Q3)	1.6 (0.7–3.4), *n* = 156	4.6 (1.6–7.8), *n* = 156	***p*** < **0.001**	*r* = −0.56
∆ CRP Day 5 (mg/dL), median (Q1–Q3)	0.8 (0.2–1.9), *n* = 32	3.7 (1.6–6.6), *n* = 37	***p*** = **0.031**	*r* = −0.90
∆ WBC 6 h (×10⁹/L), median (Q1–Q3)	3.8 (1.8–5.8), *n* = 160	4.5 (2.7–6.0), *n* = 160	***p*** = **0.041**	*r* = −0.17
∆ WBC Day 3 (×10⁹/L) median (Q1–Q3)	1.3 (0.1–2.5), *n* = 156	1.3 (0.1–2.4), *n* = 156	*p* = 0.417	*r* = 0.07
∆ WBC Day 5 (×10⁹/L), median (Q1–Q3)	0.9 (0.2–2.1), *n* = 31	0.3 (−0.3–1.2), *n* = 37	*p* = 0.055	*r* = 0.90
Haemoglobin decrease (g/dL) median (Q1–Q3)	1.5 (1.1–2.0), *n* = 161	1.9 (1.4–2.4), *n* = 161	***p*** < **0.001**	*r* = −0.39
Calculated blood loss (mL), median (Q1–Q3)	532 (364–714), *n* = 161	669 (499–889), *n* = 161	***p*** < **0.001**	*r* = −0.34

*Note*: Bold values was considered statistically significant at *p*‐value ≤ 0.05.

Abbreviations: CRP, C‐reactive protein; TKA, total knee arthroplasty; UKA, unicompartmental knee arthroplasty; WBC, white blood cell count.

**Figure 3:**
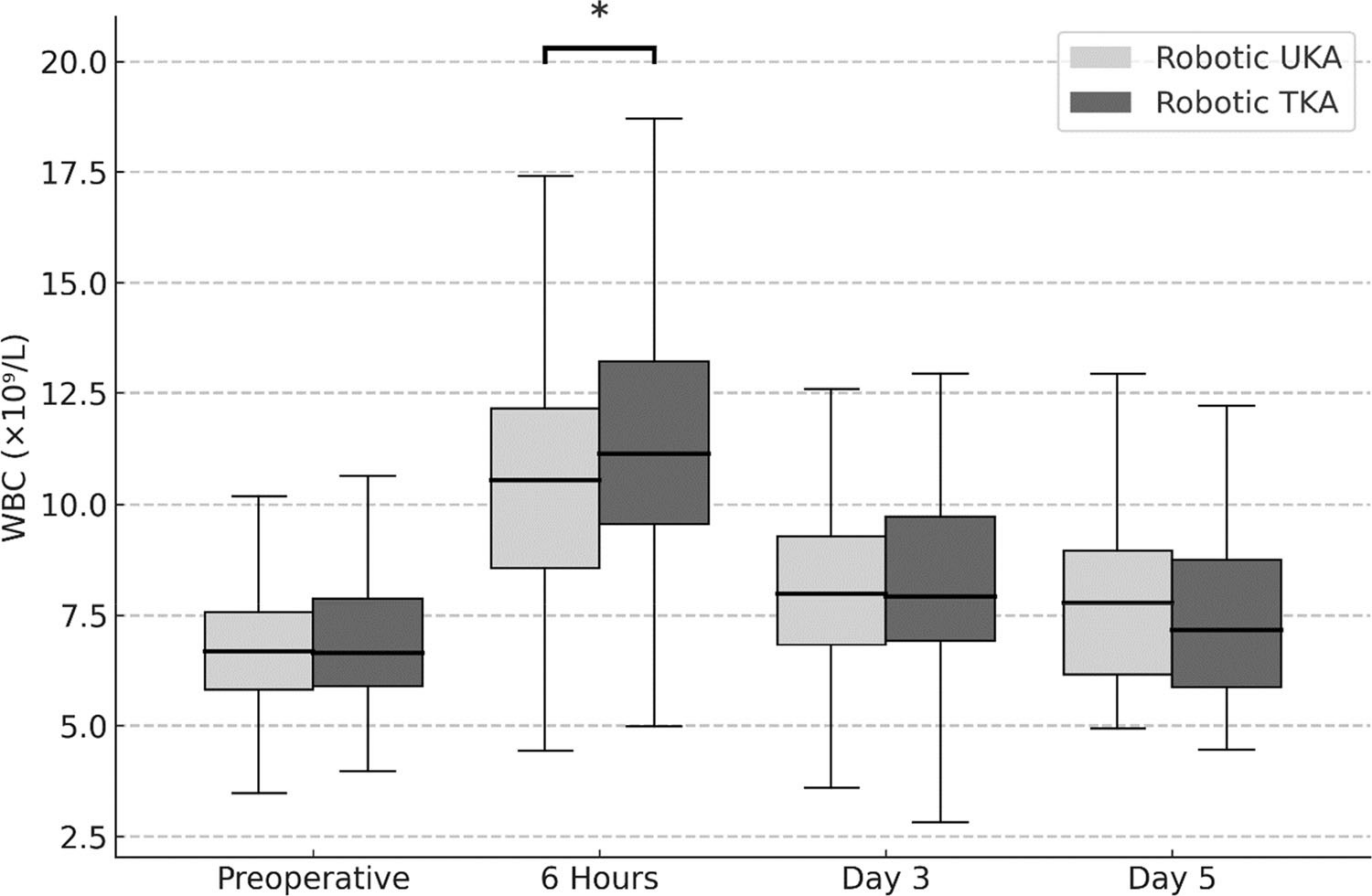
White blood cell count (WBC) levels (median, ×10⁹/L) over time in robotic unicompartmental knee arthroplasty (raUKA) and total knee arthroplasty (raTKA). *p < 0.05. For the statistical analysis, postoperative WBC values were adjusted by subtracting the preoperative baseline value.

WBC counts reached their maximum at 6 h postoperatively in both groups and declined thereafter. At 6 h, WBC counts were significantly lower in the raUKA group, whereas no significant differences were observed between groups on postoperative Days 3 or 5 (Figure 3, Table 2).

**Figure 4:**
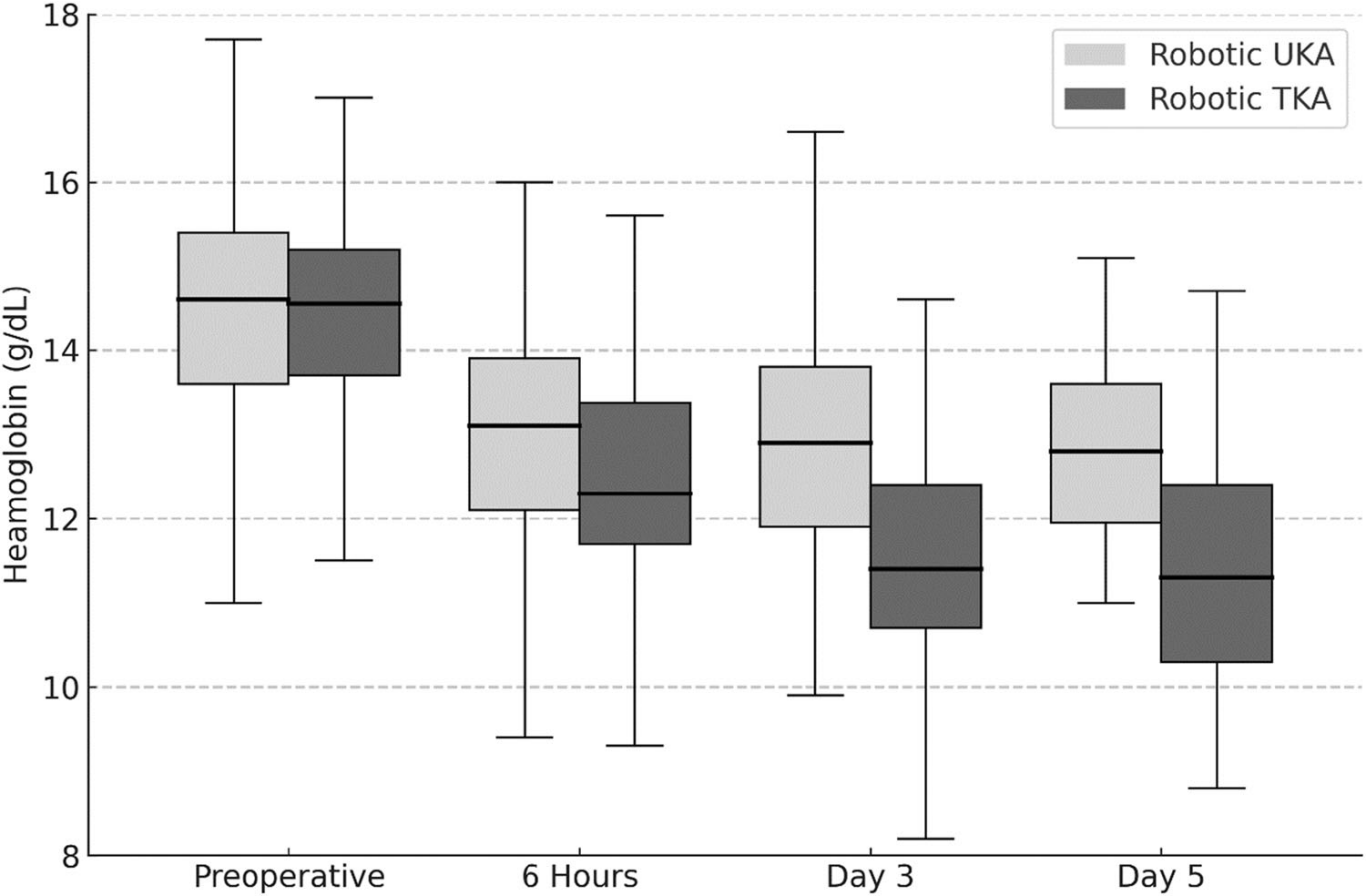
Haemoglobin (Hb) levels (median, g/dL) over time in robotic unicompartmental knee arthroplasty (raUKA) and total knee arthroplasty (raTKA). The decrease in Hb was calculated as the difference between preoperative and postoperative values, and the resulting ΔHb was used for statistical analysis.

The median operative time was significantly shorter in the raUKA group, with a median difference of 14 minutes compared with raTKA. Haemoglobin decrease and calculated intraoperative blood loss were also significantly lower in the raUKA group. One patient in the raTKA group required a blood transfusion, whereas none were required in the raUKA group. The lowest postoperative haemoglobin levels were observed on postoperative Day 5 in both groups (Figure 4, Table 2).

## DISCUSSION

This study provides the first characterisation of the systemic inflammatory response after raUKA. It was found that raUKA was associated with significantly lower postoperative CRP levels, reduced blood loss, and shorter operative times compared with raTKA. These findings establish valuable reference data for clinical monitoring and may help explain the superior early recovery consistently reported after raUKA.

CRP is widely used to track postoperative inflammation and detect complications after arthroplasty [39]. Consistent with prior reports, CRP peaked on postoperative days 2–3 [15, 33, 34, 38] and normalised within 2–4 weeks [15, 34]. In our cohort, CRP levels on Days 3 and 5 were significantly lower in the raUKA group than in the raTKA group. Because CRP elevation reflects the magnitude of surgical trauma [32], particularly the extent of bone resection [22], this difference likely reflects the less invasive nature of raUKA.

Prior studies of conventional UKA have consistently shown lower postoperative CRP levels compared with TKA [1, 19, 37, 42]. Interestingly, the absolute CRP values reported in most conventional UKA studies were higher than those observed in our raUKA cohort [1, 22, 37, 42]. Whether this reflects a true advantage of robotic assistance, or instead differences in patient selection or measurement methods, remains uncertain.

In contrast, WBC counts differed only at 6 h postoperatively, with no differences on Days 3 or 5. This is consistent with previous reports [30] and confirms that WBC is a less sensitive marker of surgical trauma than CRP. Interestingly, CRP levels after raTKA in this cohort were slightly higher than in our prior series [30], which may reflect the higher proportion of male patients, as men are known to exhibit greater CRP elevations after TKA [1, 44].

Blood loss and haemoglobin decline were also significantly lower in raUKA, corroborating earlier studies of both robotic and conventional UKA [17, 28, 36]. Importantly, haemoglobin nadirs occurred on postoperative Day 5 in both groups, a clinically relevant observation in fast‐track settings where patients are often discharged earlier. This delayed decline suggests a risk of underestimating anaemia at discharge. Within raUKA systems, Leelasestaporn et al. reported lower blood loss with the Mako® platform compared with the Navio™ platform [23], indicating that platform‐specific differences may also influence perioperative outcomes.

Operative time was shorter for raUKA than for raTKA, consistent with evidence from manual UKA studies [7]. Taken together, reduced inflammation, less blood loss, and shorter operative times may contribute to the faster recovery and superior early outcomes consistently reported after both robotic and conventional UKA compared with TKA [10, 18, 20, 26, 40, 41, 43]. These perioperative advantages are particularly relevant in elderly patients, where UKA has demonstrated benefits even in those over 75 [11] and 80 years of age [7]. Our findings support raUKA as a valuable option in this population, where minimising surgical trauma is critical.

### Limitations

This study has several limitations. First, its retrospective design carries an inherent risk of bias, although robust propensity score matching was applied to minimise confounding. Second, fewer patients were available for analysis on postoperative Day 5 due to early discharge, which may have affected the observed biomarker trajectories. Finally, comparisons with conventional UKA were not possible, as only robotic UKA has been performed at our institution since 2019.

### Clinical relevance

Understanding the biological impact of raUKA compared with raTKA is clinically important, as it highlights the physiological mechanisms underlying faster recovery after partial knee replacement. This study characterises the systemic inflammatory response following raUKA, demonstrating lower postoperative CRP levels, reduced blood loss, and shorter operative times. These findings support raUKA as a less invasive surgical option and provide biomarker reference values that may guide perioperative management, patient selection, and counselling.

## CONCLUSION

This study defines the postoperative inflammatory trajectory after raUKA, showing that CRP and WBC levels follow a lower and more favourable course than after raTKA. The presented trajectories offer practical reference values for clinical monitoring. Overall, these data reinforce raUKA as a less invasive alternative to raTKA and support its growing role in appropriately selected patients.

## AUTHOR CONTRIBUTIONS

**Dirk Müller**: Conceptualisation; methodology; data collection; analysis, interpretation of patient data and statistics; writing—original draft preparation. **Florian Pohlig**: Conceptualisation and methodology; analysis; interpretation of patient data and statistics; writing—review and editing. **Benjamin Schloßmacher**: Writing—review and editing. **Vincent Lallinger**: Writing—review and editing. **Michael T. Hirschmann**: Writing—review and editing. **Rüdiger von Eisenhart‐Rothe**: Writing—review and editing. **Igor Lazic**: Analysis; interpretation of patient data and statistics; writing—review and editing. All authors have read and approved the final version of the manuscript.

## CONFLICT OF INTEREST STATEMENT

The authors declare no conflicts of interest.

## ETHICS STATEMENT

Ethical approval was granted by the local Ethics Committee of Technical University of Munich (IRB approval number 714/20 S). Informed consent was obtained from all patients prior to study inclusion.

## Data Availability

The data sets generated and/or analysed during the current study are available from the corresponding author on reasonable request.

## References

[1] Altinayak H , Karatekin YS , Balta O . The relationship of C‐reaktif protein level after knee artroplasty with gender difference and type of artroplasty. J Orthop Surg. 2023;31:10225536231190309. 10.1177/10225536231190309 37501564

[2] Batailler C , White N , Ranaldi FM , Neyret P , Servien E , Lustig S . Improved implant position and lower revision rate with robotic‐assisted unicompartmental knee arthroplasty. Knee Surg Sports Traumatol Arthrosc. 2019;27:1232–1240. 30066017 10.1007/s00167-018-5081-5

[3] Bell SW , Anthony I , Jones B , MacLean A , Rowe P , Blyth M . Improved accuracy of component positioning with robotic‐assisted unicompartmental knee arthroplasty: data from a prospective, randomized controlled study. J Bone Joint Surg Am. 2016;98:627–635. 27098321 10.2106/JBJS.15.00664

[4] Clement ND , Bell A , Simpson P , Macpherson G , Patton JT , Hamilton DF . Robotic‐assisted unicompartmental knee arthroplasty has a greater early functional outcome when compared to manual total knee arthroplasty for isolated medial compartment arthritis. Bone Joint Res. 2020;9:15–22. 32435451 10.1302/2046-3758.91.BJR-2019-0147.R1 PMC7229306

[5] Cobb J , Henckel J , Gomes P , Harris S , Jakopec M , Rodriguez F , et al. Hands‐on robotic unicompartmental knee replacement. J Bone Joint Surg Br. 2006;88–B:188–197. 10.1302/0301-620X.88B2.17220 16434522

[6] Cool CL , Needham KA , Khlopas A , Mont MA . Revision analysis of robotic arm‐assisted and manual unicompartmental knee arthroplasty. J Arthroplasty. 2019;34:926–931. 31010509 10.1016/j.arth.2019.01.018

[7] D'ambrosi R , Ursino C , Mariani I , Ursino N , Formica M , Chen AF . Clinical outcomes, complications, and survivorship for unicompartmental knee arthroplasty versus total knee arthroplasty in patients aged 80 years and older with isolated medial knee osteoarthritis: a matched cohort analysis. Arch Orthop Trauma Surg. 2023;143:6371–6379. 37244888 10.1007/s00402-023-04916-9 PMC10491502

[8] Deirmengian GK , Zmistowski B , Jacovides C , O'neil J , Parvizi J . Leukocytosis is common after total hip and knee arthroplasty. Clin Orthop Relat Res. 2011;469:3031–3036. 21461866 10.1007/s11999-011-1887-x PMC3183208

[9] Diquattro E , Lettner J , Adriani M , Prill R , Salzmann M , Becker R . High accuracy of component positioning and restoration of lower limb alignment using robotic medial unicompartmental knee arthroplasty. Knee Surg Sports Traumatol Arthrosc. 2025;33:1982–1991. 39369428 10.1002/ksa.12484 PMC12104785

[10] Drager J , Hart A , Khalil JA , Zukor DJ , Bergeron SG , Antoniou J . Shorter hospital stay and lower 30‐day readmission after unicondylar knee arthroplasty compared to total knee arthroplasty. J Arthroplasty. 2016;31:356–361. 26476471 10.1016/j.arth.2015.09.014

[11] Fabre‐Aubrespy M , Ollivier M , Pesenti S , Parratte S , Argenson JN . Unicompartmental knee arthroplasty in patients older than 75 results in better clinical outcomes and similar survivorship compared to total knee arthroplasty. a matched controlled study. J Arthroplasty. 2016;31:2668–2671. 27480824 10.1016/j.arth.2016.06.034

[12] Gilmour A , MacLean AD , Rowe PJ , Banger MS , Donnelly I , Jones BG , et al. Robotic‐arm–assisted vs conventional unicompartmental knee arthroplasty. the 2‐year clinical outcomes of a randomized controlled trial. J Arthroplasty. 2018;33:S109–S115. 29627257 10.1016/j.arth.2018.02.050

[13] Good L , Peterson E , Lisander B . Tranexamic acid decreases external blood loss but not hidden blood loss in total knee replacement. Br J Anaesth. 2003;90:596–599. 12697586 10.1093/bja/aeg111

[14] Herry Y , Batailler C , Lording T , Servien E , Neyret P , Lustig S . Improved joint‐line restitution in unicompartmental knee arthroplasty using a robotic‐assisted surgical technique. Int Orthop. 2017;41:2265–2271. 28913557 10.1007/s00264-017-3633-9

[15] Huang ZY , Huang Q , Wang LY , Lei YT , Xu H , Shen B , et al. Normal trajectory of Interleukin‐6 and C‐reactive protein in the perioperative period of total knee arthroplasty under an enhanced recovery after surgery scenario. BMC Musculoskelet Disord. 2020;21:264. 32316949 10.1186/s12891-020-03283-5 PMC7175526

[16] Kayani B , Konan S , Tahmassebi J , Rowan FE , Haddad FS . An assessment of early functional rehabilitation and hospital discharge in conventional versus robotic‐arm assisted unicompartmental knee arthroplasty: a prospective cohort study. Bone Joint J. 2019;101–b:24–33. 10.1302/0301-620X.101B1.BJJ-2018-0564.R2 30601042

[17] Khan H , Dhillon K , Mahapatra P , Popat R , Zakieh O , Kim WJ , et al. Blood loss and transfusion risk in robotic‐assisted knee arthroplasty: a retrospective analysis. Int J Med Robot + Comput Assist Surg. 2021;17:e2308. 10.1002/rcs.2308 34288356

[18] Kievit AJ , Kuijer P , de Haan LJ , Koenraadt KLM , Kerkhoffs G , Schafroth MU , et al. Patients return to work sooner after unicompartmental knee arthroplasty than after total knee arthroplasty. Knee Surg Sports Traumatol Arthrosc. 2020;28:2905–2916. 31471724 10.1007/s00167-019-05667-0 PMC7471109

[19] Kılıçarslan K , Naldöven ÖF , Veizi E , Güven Ş , Çepni Ş , Fırat A . C‐reactive protein and erythrocyte sedimentation rates after total and unicompartmental knee arthroplasty‐less implant equals quicker normalization. J Long Term Eff Med Implants. 2024;34:49–55. 10.1615/JLongTermEffMedImplants.2023050965 38842232

[20] Kleeblad LJ , van der List JP , Zuiderbaan HA , Pearle AD . Larger range of motion and increased return to activity, but higher revision rates following unicompartmental versus total knee arthroplasty in patients under 65: a systematic review. Knee Surg Sports Traumatol Arthrosc. 2018;26:1811–1822. 29185005 10.1007/s00167-017-4817-y

[21] Kwon SC , Jung HJ , Lee JH , Hyun JT , Hwang JH , Kim JI . Robotic‐assisted medial unicompartmental knee arthroplasty restored prearthritic alignment and led to superior functional outcomes compared with conventional techniques. Knee Surg Sports Traumatol Arthrosc. 2025;33:265–273. 38796719 10.1002/ksa.12278

[22] Larsson S , Thelander U , Friberg S . C‐reactive protein (CRP) levels after elective orthopedic surgery. Clin Orthop Relat Res. 1992;237–242. 1735220

[23] Leelasestaporn C , Tarnpichprasert T , Arirachakaran A , Kongtharvonskul J . Comparison of 1‐year outcomes between MAKO versus NAVIO robot‐assisted medial UKA: nonrandomized, prospective, comparative study. Knee Surg Relat Res. 2020;32:13. 32660619 10.1186/s43019-020-00030-x PMC7219215

[24] Lonner JH , John TK , Conditt MA . Robotic arm‐assisted UKA improves tibial component alignment: a pilot study. Clin Orthop Relat Res. 2010;468:141–146. 19593669 10.1007/s11999-009-0977-5 PMC2795844

[25] Lustig S , Sappey‐Marinier E , Fary C , Servien E , Parratte S , Batailler C . Personalized alignment in total knee arthroplasty: current concepts. SICOT‐J. 2021;7:19. 33812467 10.1051/sicotj/2021021 PMC8019550

[26] Manara JR , Nixon M , Tippett B , Pretty W , Collopy D , Clark GW . A case‐matched series comparing functional outcomes for robotic‐assisted unicompartmental knee arthroplasty versus functionally aligned robotic‐assisted total knee arthroplasty. Bone Joint Open. 2024;5:1123–1129. 39701140 10.1302/2633-1462.512.BJO-2024-0086.R2 PMC11658844

[27] Mergenthaler G , Batailler C , Lording T , Servien E , Lustig S . Is robotic‐assisted unicompartmental knee arthroplasty a safe procedure? A case control study. Knee Surg Sports Traumatol Arthrosc. 2021;29:931–938. 32390119 10.1007/s00167-020-06051-z

[28] Migliorini F , Tingart M , Niewiera M , Rath B , Eschweiler J . Unicompartmental versus total knee arthroplasty for knee osteoarthritis. Eur J Orthop Surg Traumatol. 2019;29:947–955. 30535643 10.1007/s00590-018-2358-9

[29] Motesharei A , Rowe P , Blyth M , Jones B , Maclean A . A comparison of gait one year post operation in an RCT of robotic UKA versus traditional Oxford UKA. Gait Posture. 2018;62:41–45. 29524796 10.1016/j.gaitpost.2018.02.029

[30] Müller D , Lazic I , Schloßmacher B , Lallinger V , Hirschmann MT , von Eisenhart‐Rothe R , et al. Robotic arm‐assisted total knee arthroplasty reduces postoperative inflammatory response and blood loss compared to manual total knee arthroplasty: a matched‐pairs analysis of 688 patients. Knee Surg Sports Traumatol Arthrosc. 2026;34(5):1691–1699. 10.1002/ksa.70054 PMC13122751 40923399

[31] Nadler SB , Hidalgo JH , Bloch T . Prediction of blood volume in normal human adults. Surgery. 1962;51:224–232. 21936146

[32] Neumaier M , Metak G , Scherer MA . C‐reactive protein as a parameter of surgical trauma: CRP response after different types of surgery in 349 hip fractures. Acta Orthop. 2006;77:788–790. 17068712 10.1080/17453670610013006

[33] Niskanen RO , Korkala O , Pammo H . Serum C‐reactive protein levels after total hip and knee arthroplasty. J Bone Joint Surg Br. 1996;78:431–433. 8636181

[34] Oelsner WK , Engstrom SM , Benvenuti MA , An TJ , Jacobson RA , Polkowski GG , et al. Characterizing the acute phase response in healthy patients following total joint arthroplasty: predictable and consistent. J Arthroplasty. 2017;32:309–314. 27554779 10.1016/j.arth.2016.06.027 PMC7252910

[35] Rivière C , Sivaloganathan S , Villet L , Cartier P , Lustig S , Vendittoli PA , et al. Kinematic alignment of medial UKA is safe: a systematic review. Knee Surg Sports Traumatol Arthrosc. 2022;30:1082–1094. 33743031 10.1007/s00167-021-06462-6

[36] Schwab PE , Lavand'homme P , Yombi JC , Thienpont E . Lower blood loss after unicompartmental than total knee arthroplasty. Knee Surg Sports Traumatol Arthrosc. 2015;23:3494–3500. 25063489 10.1007/s00167-014-3188-x

[37] Sereda AP , Rukina AN , Trusova YV , Dzhavadov AA , Cherny AA , Bozhkova SA , et al. Dynamics of C‐reactive protein level after orthopedic surgeries. J Orthop. 2024;47:1–7. 38046451 10.1016/j.jor.2023.11.014 PMC10689206

[38] Shen H , Zhang N , Zhang X , Ji W . C‐reactive protein levels after 4 types of arthroplasty. Acta Orthop. 2009;80:330–333. 19593724 10.3109/17453670903066596 PMC2823221

[39] Sigmund IK , Puchner SE , Windhager R . Serum inflammatory biomarkers in the diagnosis of periprosthetic joint infections. Biomedicines. 2021;9:1128. 34572314 10.3390/biomedicines9091128 PMC8467465

[40] Suarez JC , Saxena A , Arguelles W , Watson Perez JM , Ramamoorthy V , Hernandez Y , et al. Unicompartmental knee arthroplasty vs total knee arthroplasty: a risk‐adjusted comparison of 30‐day outcomes using National Data From 2014 to 2018. Arthroplast Today. 2022;17:114–119. 36082284 10.1016/j.artd.2022.06.017 PMC9445223

[41] Sullivan GE , Highland KB , Booth GJ , Dunnum AP , Goldman AH . The relationship between age and 30‐day outcomes following unicompartmental versus total knee arthroplasty. J Arthroplasty. 2025;40:611–618. 39233099 10.1016/j.arth.2024.08.053

[42] Thienpont E , Grosu I , Jonckheere S , Yombi JC . C‐reactive protein (CRP) in different types of minimally invasive knee arthroplasty. Knee Surg Sports Traumatol Arthrosc. 2013;21:2603–2610. 23274268 10.1007/s00167-012-2345-3

[43] Tripathy SK , Varghese P , Srinivasan A , Goyal T , Purudappa PP , Sen RK , et al. Joint awareness after unicompartmental knee arthroplasty and total knee arthroplasty: a systematic review and meta‐analysis of cohort studies. Knee Surg Sports Traumatol Arthrosc. 2021;29:3478–3487. 33078218 10.1007/s00167-020-06327-4

[44] Windisch C , Brodt S , Roehner E , Matziolis G . The C‐reactive protein level after total knee arthroplasty is gender specific. Knee Surg Sports Traumatol Arthrosc. 2016;24:3163–3167. 27535675 10.1007/s00167-016-4289-5

[45] Zhang J , Ng N , Scott CEH , Blyth MJG , Haddad FS , Macpherson GJ , et al. Robotic arm‐assisted versus manual unicompartmental knee arthroplasty. Bone Joint J. 2022;104–B:541–548. 10.1302/0301-620X.104B5.BJJ-2021-1506.R1 PMC9948441 35491572

